# The association between caregiver education on adult T2DM and patient’s outcomes in community: A systematic review

**DOI:** 10.1093/geroni/igab046.2944

**Published:** 2021-12-17

**Authors:** Aluem Tark, Jiyoun Song, Jeong-Yeon Kim, So Yeon Park, Kyungmi Woo

**Affiliations:** 1 University of Iowa, University of Iowa, Iowa, United States; 2 Columbia University, Columbia University, New York, United States; 3 Seoul National University, Seoul, Seoul-t'ukpyolsi, Republic of Korea

## Abstract

Introduction Adult type 2 diabetes (T2DM) threatens public health and most patients manage their diabetic condition while in the community. As it is challenging for patients to properly manage diabetes alone, caregiver involvement in T2DM patient care is encouraged. This study aimed to examine the association between caregiver involvement in T2DM education within a community and the patients’ diabetes care outcomes (e.g., glycated hemoglobin (HbA1c) level, behavior, or hospitalization). Methods The available scientific literature in PubMed, Cochrane, EMBASE, and CINAHL was searched. The methodological quality of bias was assessed using the Cochrane risk of bias tool. Results A total of 13 out of 741 published studies were synthesized in this review. There is evidence that caregiver involvement in T2DM education is effective in the reduction of HbA1C and BMI, but not necessarily effective in reducing lipids. Study results indicate that caregiver related interventions can significantly improve patient diabetes knowledge, physical activity, and self-efficacy, but results were more mixed regarding medication adherence. Risk of bias analysis classified the majority of studies (77%) to be moderate or high quality. Conclusion This review aimed to explore the association between caregiver involvement in adult T2DM education in the community and patients’ diabetes care outcomes. The findings show an improvement in biological and behavioral self-management outcomes with caregivers involved in T2DM education, though no studies examined the direct association between complications or hospital readmission. Future research focused on tailored interventions and longer follow-up of patient outcomes are recommended.

